# Visualization of the auditory pathway in rats with ^18^F-FDG PET activation studies based on different auditory stimuli and reference conditions including cochlea ablation

**DOI:** 10.1371/journal.pone.0205044

**Published:** 2018-10-02

**Authors:** Martin Mamach, Mariella Kessler, Jens P. Bankstahl, Florian Wilke, Lilli Geworski, Frank M. Bengel, Simone Kurt, Georg Berding

**Affiliations:** 1 Department of Medical Physics and Radiation Protection, Hannover Medical School, Hannover, Germany; 2 Cluster of Excellence Hearing4all, Hannover Medical School, Hannover, Germany; 3 Department of Nuclear Medicine, Hannover Medical School, Hannover, Germany; 4 Department of Otolaryngology, Hannover Medical School, Hannover, Germany; 5 Department of Biophysics, Center for Integrative Physiology and Molecular Medicine CIPMM, Saarland University, Homburg, Germany; Universidad de Salamanca, SPAIN

## Abstract

Activation studies with positron emission tomography (PET) in auditory implant users explained some of the mechanisms underlying the variability of achieved speech comprehension. Since future developments of auditory implants will include studies in rodents, we aimed to inversely translate functional PET imaging to rats. In normal hearing rats, activity in auditory and non-auditory regions was studied using ^18^F-fluorodeoxyglucose (^18^F-FDG) PET with 3 different acoustic conditions: sound attenuated laboratory background, continuous white noise and rippled noise. Additionally, bilateral cochlea ablated animals were scanned. 3D image data were transferred into a stereotaxic standard space and evaluated using volume of interest (VOI) analyses and statistical parametric mapping (SPM). In normal hearing rats alongside the auditory pathway consistent activations of the nucleus cochlearis (NC), olivary complex (OC) and inferior colliculus (IC) were seen comparing stimuli with background. In this respect, no increased activation could be detected in the auditory cortex (AC), which even showed deactivation with white noise stimulation. Nevertheless, higher activity in the AC in normal hearing rats was observed for all 3 auditory conditions against the cochlea ablated status. Vice versa, in ablated status activity in the olfactory nucleus (ON) was higher compared to all auditory conditions in normal hearing rats. Our results indicate that activations can be demonstrated in normal hearing animals based on ^18^F-FDG PET in nuclei along the central auditory pathway with different types of noise stimuli. However, in the AC missing activation with respect to the background advises the need for more rigorous background noise attenuation for non-invasive reference conditions. Finally, our data suggest cross-modal activation of the olfactory system following cochlea ablation–underlining, that ^18^F-FDG PET appears to be well suited to study plasticity in rat models for cochlear implantation.

## Introduction

Since their first clinical application in the early 1960s cochlear implants (CI) enabled a remarkable success story in the treatment of inner ear deafness [1]. Nevertheless, the variability of individual performance in speech comprehension after CI implantation is not fully understood. In 30% of the cases, despite early implantation in childhood, the expected hearing performance is not achieved–without explanation in 78% of these [2–4]. In this context, functional neuroimaging after implantation using positron emission tomography (PET) improved already the pathophysiologic understanding of underlying plasticity in specialized auditory regions as well as at the level of neuronal circuits required for a successful outcome [5–7]. Nevertheless, since it is complicated to address some of the basic factors related to the success of cochlear implantation in patients, like it is done e.g. in molecular biological / proteomics research, animal models and related diagnostic approaches are required. Specifically, cochlear implantation has been implemented in rats–chosen due to their low mortality rate following surgical intervention and the extensive auditory research already performed in this species [8, 9]. With respect to diagnostic approaches for rat brain imaging, PET achieved sufficient spatial resolution with the development of dedicated small animal scanners about 15 years ago [10]. Subsequently, specific tools e.g. stereotaxic VOI atlases for spatial assignment of specific effects have been developed [11, 12]. For auditory research, this technique has mostly been used employing ^18^F-2-fluoro-2-deoxy-D-glucose (^18^F-FDG) as a marker of neuronal activity. Auditory and visual stimuli as well as cochlea ablation and tinnitus condition have been investigated [8, 13–15]. However, these were pioneering studies, demonstrating the general potential of the methodology, but including shortcomings with respect to the development of ^18^F-FDG PET as a standardized quantitative diagnostic method to serve in the further development of treatment options for hearing loss in particular via rat models of cochlear implantation. Shortcomings include for example: (i) no consideration of adaptation to stimulation paradigms, (ii) lack of standardization of environment with background or reference conditions, (iii) lack of comparison to bilateral complete hearing loss as absolute reference in order to estimate observed effects sizes and (iv) no exclusion / proof of effects due to auditory stimulation in non-auditory regions (including potential cross-modal activations).

To address these questions, we (i) used refined stimuli designed to avoid adaptation which were previously employed in electrophysiology [16], (ii) utilized a sound attenuating box as environmental standardization and tested with 2 possible auditory baseline conditions, (iii) added longitudinal tests after bilateral cochlea ablation as physiological baseline for a subset of animals and (iv) implemented a volume of interest (VOI) template for automated spatial assignment and relative quantification of effects in 5 essential brain structures of the auditory system and additionally 3 reference brain regions.

## Materials and methods

This study was approved by the Lower Saxony State Office for Consumer Protection and Food Safety (LAVES, ID: 33.14-42502-04-14/1625) in accordance with German and European regulations for animal experiments. Part of this approval was validation by an ethics committee of the LAVES. This study was designed and is reported in accordance with the ARRIVE guidelines [17].

### Animals

In this study 12 healthy, normal hearing, young adult, female Sprague-Dawley rats (Charles River, Sulzfeld, Germany) in 2 groups of 6 animals were included. The animals were 16 weeks old when bought and were habituated to the laboratory environment for 1 week and trained for 3 additional weeks for habituation to the acoustic stimulation setup. All animals were housed in pairs in individually ventilated cages with (50±5) % humidity at (22±1) °C with 14–10 hours day-night cycle. They had ad libitum access to autoclaved tap water and a standard laboratory diet (1324 TPF, Altromin, Lage, Germany). The rats (weight 289 g ± 19 g) were scanned at ages 20–39 weeks.

### Experimental procedure

**Fig 1:**
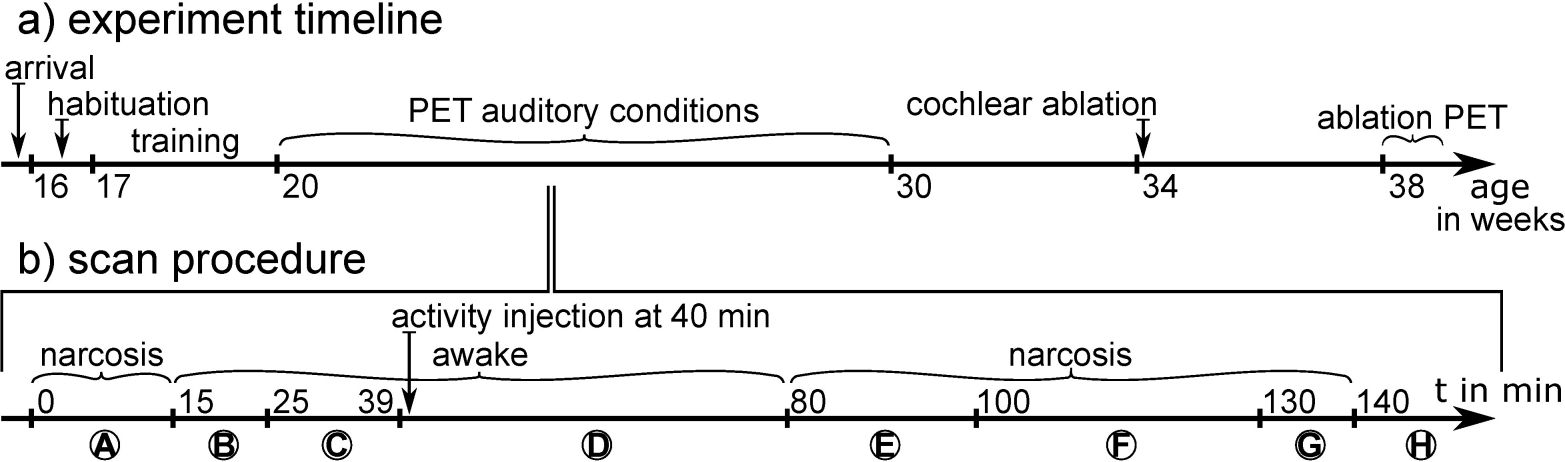
**Timelines for a) general experimental setup and b) a single scan procedure.** The scan procedure includes (A) placement of the tail vein catheter and insertion of the rat in the restriction tube into the box, (B) wake-up period, (C) adaption period to sound deprivation, (D) acoustic conditions, (E) preparation for scan, (F) PET scan, (G) CT scan and (H) the return to the home cage.

**Fig 2:**
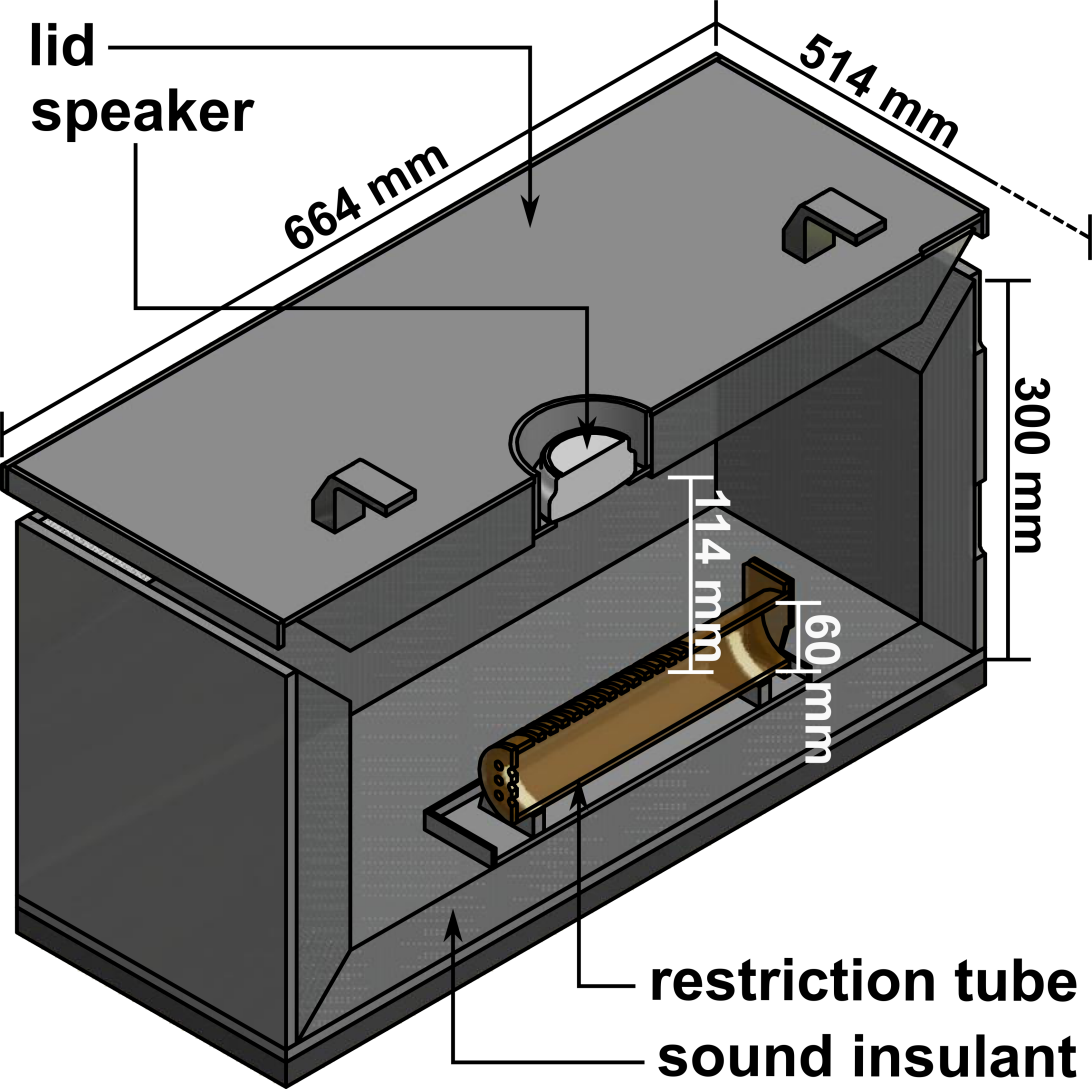
Schematic view of the custom-made sound-attenuating box. The speaker is placed in the cover lid. Holes in the front, top and end of the animal restriction tube allowed respectively for sufficient ventilation, sound permeability and access to the tail of the animals.

Fig 1A) illustrates the general experimental setup in a timeline. Fig 1B) shows the timing of the applied acoustic conditions, tracer injection and subsequent ^18^F-FDG PET imaging. The animals were shortly anesthetized with an isoflurane (FORENE, AbbVie, Wiesbaden, Germany) in humidified oxygen for placement of an intravenous (i.v.) catheter into a tail vein and afterwards placed into the restriction tube into the sound attenuation box. The anesthesia was initialized with 3% isoflurane concentration and a flow of 3 l/min. After a wake-up period of 10 min, the box (Fig 2) was fully closed and the sound level reduced to unavoidable background noises for 14 min. Subsequently, an auditory condition was started lasting for 41 min. 60 sec after the start, ^18^F-FDG was applied as bolus injection with a closed box into the tail vein via a catheter. After the condition ended, the animals were anesthetized again with isoflurane and scanned 1 h post injection. In continuous anesthesia, the concentration of isoflurane was reduced to 1–2% and the flow to 0.8–1.0 l/min according to a target respiratory rate of 30–40 rpm. The animals were warmed during anesthesia. All animals were placed into their home cages when fully awake. Between imaging sessions, a resting period of at least 2 days was kept for each individual animal. All 12 animals were exposed to all auditory conditions. Since we could scan on average 3 animals per day, 4 subgroups were built for pseudo-randomization of the order of animals (A, B and C) and stimulation conditions (1, 2 and 3) on three scan days. On these days the order of animals and conditions complied with the following rules: (1) each animal was scanned at first, second and third position on different scanning days, (2) at each scanning day all stimulation conditions were presented at a different position. A typical sequence of animals and conditions would be: Scanning day 1: A1 B2 C3; day 2: C2 A3 B1 and day 3: B3 C1 A2. After completion of the entire study we confirmed in a statistical analysis (ANCOVA) the independence of the PET uptake values from the order of stimulus presentation (p >F: 0.5670) and order of animals (p >F: 0.5716). With respect to the status of cochlear ablation–since auditory stimulation conditions are only possible before ablation, this status had always to be performed last. After the completion of all experiments, animals were sacrificed by cervical dislocation in isoflurane anesthesia.

### Conditions

#### Stimulation environment

All auditory conditions were applied in a standardized acoustic environment consisting of a transparent motion restriction tube placed in an unlit custom made sound-attenuating box as shown in Fig 2. Opening on the top and the front of the restriction tube enable acoustic stimulation and a notch in the hatch enables access to the tail for i.v. administration. The length and diameter of the tube were designed to ensure maximal comfort for the animals after habituation training, but to avoid turning around or considerable backward of forward movement. The box reduced default laboratory background noises by 10 dB to 55 dB SPL (decibel sound pressure level) as measured with Brüel & Kjær 2636 amplifier (Brüel & Kjær, Bremen, Germany) and condenser microphone 4135 (Brüel & Kjær, Bremen, Germany) on a representative position in the restriction tube. This ensured similar acoustic conditions across all conditions via a closable opening directly above the tube indicated in Fig 2. In the lid a Wavemaster Mobi 2 speaker (Wavemaster, Bremen, Germany) was placed. The animals were observed in the closed box via an infrared camera (Syngonix 43176Y, Nürnberg, Germany) to ensure their well-being and complication-free application. The box was always placed in the same room with controlled climatic environment. All procedures were done by the same staff creating per se constant acoustic, climatic and olfactory conditions.

#### Training

As stress would affect the performance of the animals during awake acoustic stimulation, all animals were trained to this environment over 3 weeks. To familiarize the rat with the setup, they were placed in the motion restriction tube and box three times awake on subsequent days. Further habituation to waking up in the setup was performed twice under initial isoflurane anesthesia. In each training session, the animals were in the box in the tube for a 10 min wake-up period and 20 min of training conditions were applied. No catheters were applied or sham injections performed during training sessions as this was seen as unnecessary burden. Additionally, both way open tubes made of the same material with similar diameters and lengths were placed into the home cages for enrichment and habituation.

#### Auditory conditions

Three auditory conditions were selected in this study to provide different effects with respect to (i) adaptation to the stimulus and (ii) degree of activation achieved in auditory brain regions. Conditions are identified by the type of noise (two letters) and the sound pressure level in brackets.

Condition 1: Background noise—*BG(55dB)*

Condition 2: Continuous white noise—*WN(65dB)*

Condition 3: Pulsed rippled noise—*RN(95dB)*, consisting of broadband randomized frequency depending phase and amplitude modulations for 500 ms followed by 200 ms silence [16]

Proceeding from these conditions, an additional condition was constructed with slight modifications for training purposes in order to avoid adaptation effects to the auditory conditions. In order to avoid habituation to specific frequency pattern or loudness, a white noise stimulus with intermediate loudness was used in the training sessions. The purpose was to train the animals to a noisy environment without adapting to the specific stimulus. The laboratory background was used in order to train a stimulus-free environment. We alternately exposed the animals during the training to the respective conditions for a very limited duration (5 min) in comparison to the much longer exposure during FDG uptake phase for prevention of adaptation to the stimuli.

Training condition: 4 min *BG(55dB)* + 5 min *WN(75dB)* + 5 min *BG(55dB)* + 5 min *WN(75dB)* + 1 min *BG(55dB)*

All auditory stimuli were prepared using the open-source-software Audacity 2.0.5. The sound levels in dB SPL were measured using Brüel & Kjær 2636 amplifier (Brüel & Kjær, Bremen, Germany) and a condenser microphone 4135 (Brüel & Kjær, Bremen, Germany) placed within the restriction tube at an ear-representative location.

### Cochlea ablation

Bilateral cochlea ablations (*ABL*) were included as a reference of complete deprivation of the auditory system and therefore better assessment of differences in normal hearing animals due to different auditory conditions. The cochlea ablations were performed on 6 animals after their completion PET scans of the auditory conditions as described in Deutscher et al. [18]. 1 animal died after surgery resulting in 5 animals for which pairwise analysis was possible. Under anesthesia using a mix of 80 mg/kg ketamine (Ketamin, WDT, Garbsen, Germany) and 5 mg/kg xylazine (Sedaxylan, WDT, Garbsen, Germany) the skin was opened over the bulla tympanica and muscles were moved to reveal the bulla. Via a small, drill the bulla was opened and the organ of Corti destroyed with a sterile needle. An antibacterial salve (Tyrosur, Engelhard Arzneimittel, Niederdorfelden, Germany) was inserted into the bulla to avoid infections. The bulla opening was closed by applying composite glue (Tetric EvoFlow, Ivoclar Vivadent AG, Schaan, Liechtenstein), suture material (Ethicon, Johnson & Johnsen Medical GmbH, Norderstedt, Germany) and Histoacryl (B.Braun, Melsungen, Germany). To provide analgesic care, metamizole (Novaminsulfon 500mg Lichtenstein, Zentiva, Prague, Czechia) was added to the drinking water 2 days preoperative for max. 2 weeks and given subcutaneously after the operation (200 mg/kg metamizole). PET imaging was performed 3–4 weeks after ablation.

### PET/CT imaging

All rats were imaged using a hybrid Inveon PET/CT system (Siemens AG, Berlin, Germany). A dose of 18.2±0.7 MBq of the radiotracer ^18^F-FDG was injected i.v. via the lateral tail vein catheter. A 30 min PET list mode scan was started 1 h post injection and reconstructed using an OSEM3D follow by FastMAP iterative reconstruction algorithm. After two OSEM3D iterations, FastMAP generated an image of 128x128x159 voxels with a voxel size of 0.776 mm x 0.776 mm x 0.796 mm using 18 iterations and 16 subsets. A ^57^Co-transmission based attenuation correction was applied during reconstruction. PET acquisitions were followed by CT imaging using a tube voltage of 80 kV, a current of 500 μA and 120 ms exposure time of the head acquired in continuous rotation (full rotation with 180 projections) and reconstructed as filtered back projection using a Feldkamp algorithm and Shepp-Logan filter with 0.5 cutoff. The resulting CTs had a size of 512x512x512 voxels with a resolution of [0.096 mm]^3^ and were used to validate coregistration.

### Data analysis

For a regional statistical analysis on group level, brain regions were extracted using a VOI atlas for rats [12] adapted to a rat ^18^F-FDG image template [11] via corresponding MR images. Additional VOIs were drawn according to the Paxinos and Watson rat brain atlas [19]. The images were processed using PMOD3.6 software (PMOD Technologies, Zurich, Switzerland). Individual images were manually pre-aligned and followed by rigid-matching to the FDG. These spatially normalized images were then cropped to include the brain within a matrix of 93x93x120 voxels with a voxel size of [0.2 mm]^3^ as given by the template. In line with clinical methods, the image values were normalized to the observed average whole brain activity for further analysis.

We evaluated the activities in VOIs of the nucleus cochlearis (NC), the olivary complex (OC), the inferior colliculus (IC), the medial geniculate body (MGB) and the auditory cortex (AC) as auditory target regions. The somatosensory cortex (SC), cerebellum (CB) and olfactory nucleus (ON) were used as non-auditory control regions. All VOIs were analyzed in individually paired t-tests of two respective auditory conditions for all animals. All positions of coronal slides are given with reference to the ^18^F-FDG templates bregma coordinates [11]. Additionally, statistical parametric maps (SPM) including significantly activated or deactivated voxels at a threshold of p<0.001 without correction for multiple comparisons were generated based on normalized images by comparing conditions using pairwise t-testing in SPM8.0 (Wellcome Department of Cognitive Neurology, London, UK). These analyses were chosen as in preliminary analysis with p<0.05 and FEW corrections no supra-threshold voxels were found in the target region of the auditory cortex. A minimum fraction of activated voxels of 1% of the VOI was selected as extent threshold to account for random effects. The actual percentage of activated voxels in a VOI was called coverage and listed for each comparison of conditions alongside with the T_max_-values and the ratio of T_max_ to T-value at p<0.001 for assessment of extent and peak height of differences between different auditory conditions.

## Results

The used stimuli and applied reference condition determined which sub-regions of the auditory system revealed activation respectively. For individually analyzed animals activations were only observable in up to two regions, while group analyses revealed activations in up to 4 of 5 evaluated auditory regions.

### Individual analysis

**Fig 3:**
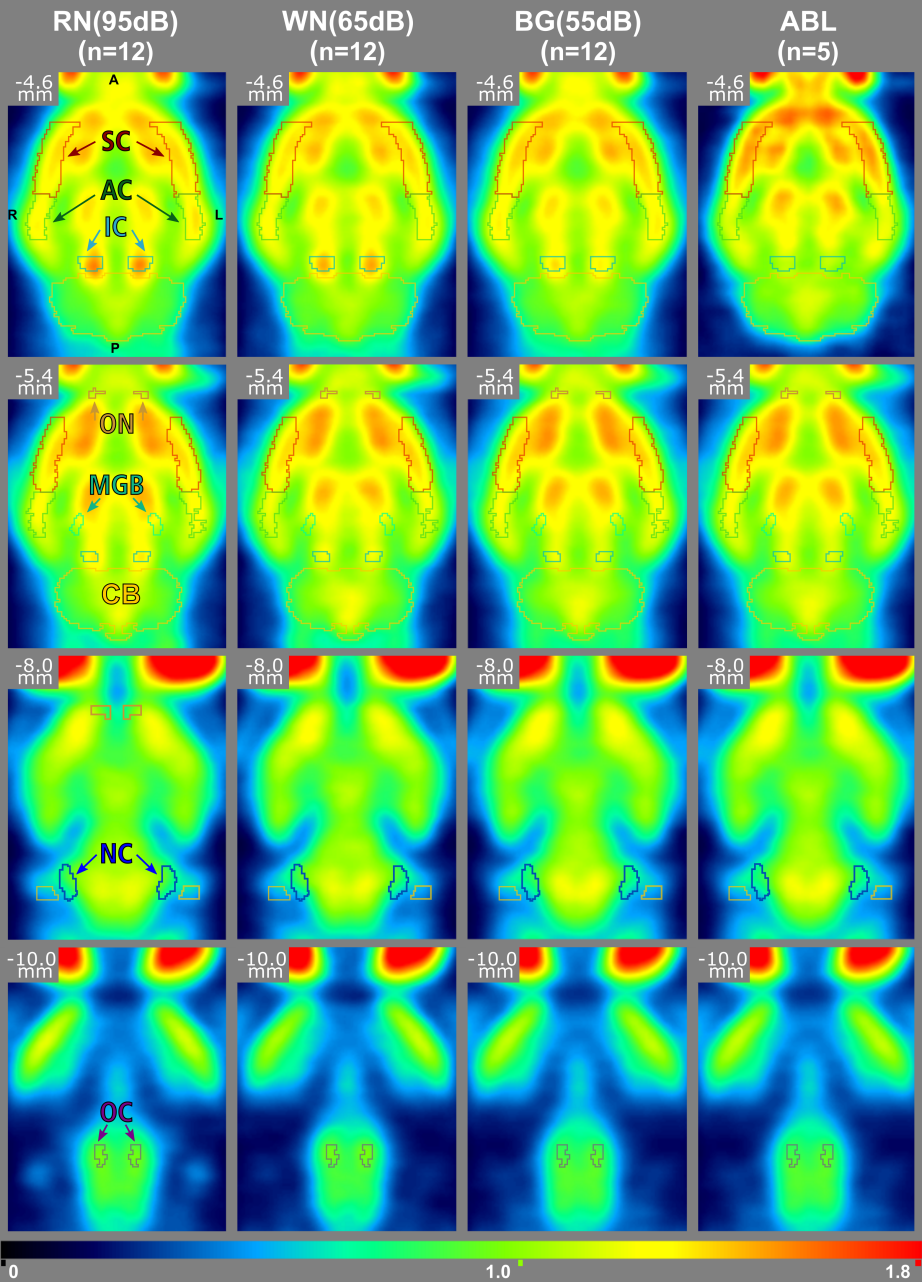
Mean normalized images for all conditions and in different coronal levels. Different activation can be visually assessed in different regions (AC–auditory cortex, IC–inferior colliculus, MGB–mediate geniculate body, OC–olivary cortex, NC–nucleus cochlearis, SC–somatosensory cortex, CB–cerebellum, ON–olfactory nucleus). The activation of the IC shown in the first two rows is strongest for the rippled noise stimulus RN(95dB) and visible with white noise WN(65dB). It is reduced to the brain mean with laboratory background BG(55dB) and below mean after ablation ABL.

In individual images, higher normalized ^18^F-FDG uptake could be visually assessed for RN(95dB) stimulation in IC and AC, while no distinct difference to other conditions was detected in MGB, NC or OC. In individual difference images of RN(95dB) and BG(55dB) which had the largest sound level difference in normal hearing animals in our experiment, visual activation evidences were limited to the IC (images not shown). Therefore group analyses were required. Fig 3 shows corresponding average images based on all individual images for each auditory condition.

### VOI analyses

In Table 1, mean percent differences in normalized uptake between stimulated and reference conditions are given for auditory and non-auditory VOIs. Significant differences in normalized uptake between conditions (“activations” or “deactivations) are indicated with grey background in Table 1.

**Table 1:** Mean and standard deviation (SD) of pairwise differences of average normalized VOI values shown for all comparisons of conditions.

	RN–BG (n = 12)		RN–WN (n = 12)		WN–BG (n = 12)		RN–ABL (n = 5)		WN–ABL (n = 5)		BG–ABL (n = 5)	
	Mean SD	p-Value	Mean SD	p-Value	Mean SD	p-Value	Mean SD	p-Value	Mean SD	p-Value	Mean SD	p-Value
AC	(0.0±2.4) %	0.9943	(2.8±3.1) %	0.0101	(-2.8±1.6) %	0.0001	(10.0±3.1) %	0.0018	(6.5±2.4) %	0.0036	(10.0±2.1) %	0.0004
IC	(15.6±3.6) %	0.0001	(3.1±7.4) %	0.1817	(12.5±6.4) %	0.0001	(35.0±3.8) %	0.0001	(27.5±5.7) %	0.0004	(18.4±4.3) %	0.0007
MGB	(6.1±3.4) %	0.0001	(5.6±3.5) %	0.0002	(0.6±2.6) %	0.4591	(8.0±7.1) %	0.0649	(2.6±6.9) %	0.4524	(1.8±6.4) %	0.5614
OC	(6.3±9.7) %	0.0445	(-0.5±6.3) %	0.7829	(6.9±9.0) %	0.0223	(20.3±9.1) %	0.0075	(17.8±9.2) %	0.0125	(8.2±6.5) %	0.0485
NC	(6.6±4.6) %	0.0004	(2.0±3.5) %	0.0761	(4.6±5.1) %	0.0091	(14.5±8.2) %	0.0168	(11.0±9.5) %	0.0600	(6.1±7.8) %	0.1572
SC	(-1.5±3.2) %	0.1278	(-1.2±3.8) %	0.2934	(-0.3±2.0) %	0.5881	(-3.1±3.4) %	0.1115	(-3.1±3.7) %	0.1684	(-2.0±2.6) %	0.1369
CB	(0.6±3.9) %	0.6053	(-0.8±4.1) %	0.5220	(1.4±2.9) %	0.1208	(1.3±3.1) %	0.4157	(1.5±2.3) %	0.2158	(-0.3±3.2) %	0.8448
ON	(0.6±6.3) %	0.7598	(1.4±5.5) %	0.3835	(-0.9±2.7) %	0.2958	(-23.4±6.7) %	0.0014	(-21.9±8.0) %	0.0037	(-20.5±7.9) %	0.0044

The corresponding p-values are shown and significances of p<0.05 are highlighted by grey shading. In normal hearing animals (RN–rippled noise, WN–white noise, BG—laboratory background), different auditory structures (AC–auditory cortex, IC–inferior colliculus, MGB–mediate geniculate body, OC–olivary cortex, NC–nucleus cochlearis) are significantly activated for different comparisons. In non-auditory regions (regions (SC–somatosensory cortex, CB–cerebellum, ON–olfactory nucleus), significant deactivation shown by the negative values are only detected comparing to ablation (ABL).

Using BG(55dB) as a reference, stimulation with either RN(95dB) or WN(65dB) resulted into significant activation in IC, OC and NC. The highest activation by (15.6±3.6) % occurred in the IC. No increase in activation could be seen with the BG(55dB) reference in the AC. Instead, stimulation by WN(65dB) induced a low but significant deactivation compared to BG(55dB) (-2.8±1.6) %, which formally introduced an increased activation, if RN(95dB) is compared to WN(65dB). Moreover, MGB activation was only induced by RN(95dB) but not with WN(65dB) stimulation. In normal hearing animals (i.e. without cochlea ablation) neither activation nor deactivation could be observed in non-auditory regions (SC, CB ON).

Employing ABL as reference condition, significant activations were observed with all other conditions (RN(95dB), WN(65dB) and BG(55dB)) in IC, OC and AC. Activations were considerably higher for all three regions–e.g. (35.0±3.8) % in the mean with RN(95dB) in IC–in comparison to those obtained with BG(55dB) as reference (15.6±3.6) %. Moreover, no activation was seen consistently in MGB–and in NC activation was only observed with RN(95dB). With respect to non-auditory regions, activity in ON was significantly higher in ABL condition compared to all acoustic conditions in healthy rats (> 20% in the mean, see Table 1 –negative algebraic sign there, for the comparison RN(95dB)—ABL indicate higher values for ABL condition). For all other non-auditory regions no activation or deactivation was found.

### SPM results

**Fig 4:**
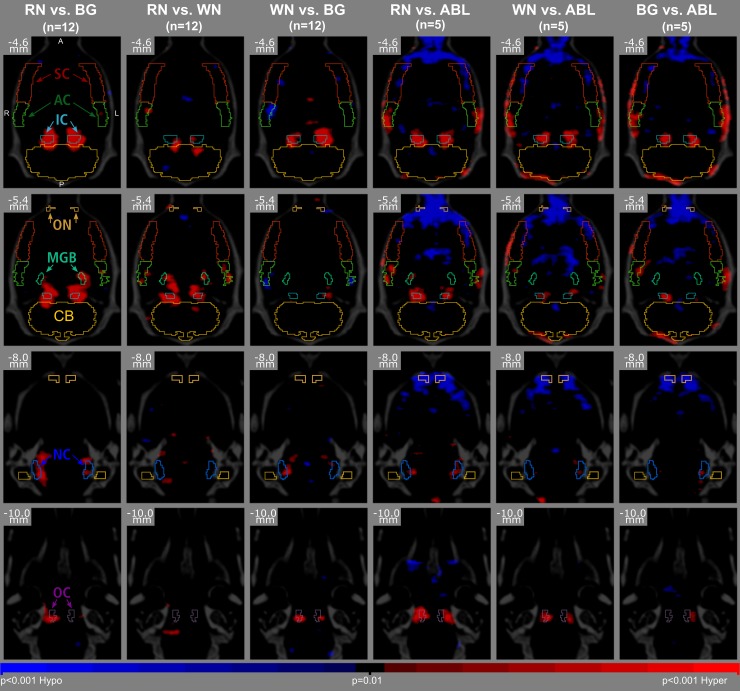
Results of voxel-wise pairwise comparison in SPM. The first 3 columns show all comparisons of auditory conditions in normal hearing animals (RN–rippled noise, WN–white noise, BG—laboratory background) and the last 3 columns show all comparisons with respect to cochlea ablation (ABL). Red shows significantly increased ^18^F-FDG uptake of the first condition versus the second, while blue shows respectively decreased uptake comparing first to second condition in the corresponding regions (AC–auditory cortex, IC–inferior colliculus, MGB–mediate geniculate body, OC–olivary cortex, NC–nucleus cochlearis, SC–somatosensory cortex, CB–cerebellum, ON–olfactory nucleus).

Corresponding to the comparisons carried out at a VOI level, pairwise voxel-based analyses were performed using SPM8.0. The results are given in Table 2 and Fig 4. They resemble by and large those obtained in VOI analyses. Maximum T-values are shown in Table 2. For improved comparability of activation strength between condition-comparisons and between anatomical regions, Table 2 contains additionally a T_max_-fraction with respect to the corresponding T-threshold for p<0.001 and the percentage of voxels significantly activated in the respective VOI (i.e. the coverage).

**Table 2:** T-values and coverage of VOIs for each condition comparison with SPM.

	RN—BG (n = 12)			RN—WN (n = 12)			WN—BG (n = 12)			RN—ABL (n = 5)			WN—ABL (n = 5)			BG—ABL (n = 5)		
	*T*_max_	$\frac{T_{\max}}{T_{p=0.001}}$	Cov.	*T*_max_	$\frac{T_{\max}}{T_{p=0.001}}$	Cov.	*T*_max_	$\frac{T_{\max}}{T_{p=0.001}}$	Cov.	*T*_max_	$\frac{T_{\max}}{T_{p=0.001}}$	Cov.	*T*_max_	$\frac{T_{\max}}{T_{p=0.001}}$	Cov.	*T*_max_	$\frac{T_{\max}}{T_{p=0.001}}$	Cov.
AC			<1%			<1%	-5.47	-1.36	3%	17.06	2.38	6%	12.09	1.69	3%	14.73	2.05	7%
IC	10.70	2.66	89%	5.43	1.35	5%	8.23	2.04	55%	33.70	4.70	67%	22.99	3.20	32%	14.39	2.01	15%
MGB	5.49	1.36	16%	5.83	1.45	14%			<1%			<1%			<1%			<1%
OC	4.77	1.19	5%			<1%	5.43	1.35	15%	13.24	1.85	12%	8.32	1.16	3%			<1%
NC	5.71	1.42	5%			<1%	5.53	1.37	2%			<1%			<1%			<1%
SC			<1%			<1%			<1%			<1%	-12.85	-1.79	1%			<1%
CB			<1%			<1%			<1%			<1%			<1%			<1%
ON			<1%			<1%			<1%	-19.58	-2.73	39%	-14.30	-1.99	26%	-13.38	-1.86	20%

(Cov.) shows the coverage of the corresponding VOIs with significant activated voxel according to SPM for all auditory regions (AC–auditory cortex, IC–inferior colliculus, MGB–mediate geniculate body, OC–olivary cortex, NC–nucleus cochlearis) and control regions (SC–somatosensory cortex, CB–cerebellum, ON–olfactory nucleus). Negative T-values indicate higher activity in the second condition (RN–rippled noise, WN–white noise, BG—laboratory background, ABL–cochlea ablation). An activation threshold of 1% of the corresponding VOI was assumed to account for random activations.

With BG(55dB) as a reference, SPM revealed in healthy animals like VOI analyses for RN or WN as stimulation condition significant activations in IC, OC and NC. Up to 89% of the IC-VOI were covered with T_max_ being 2.66 times of the T_p = 0.001_ threshold. The nearly total coverage/activation of the IC due to RN(95dB) stimulation vs. BG(55dB) is illustrated in Fig 4 by representative slices of the SPM overlaid to CT and VOI outlines (1^st^ column, 1^st^ and 2^nd^ row). Moreover, as in VOI analyses, SPM showed a deactivation of the AC under WN(65dB) stimulation compared to BG(55dB) (coverage 3%) and MGB activation induced by RN(95dB) but not with WN(65dB) stimulation. Bilateral deactivation in the AC (due to WN(65dB) stimulus against BG(55dB)) can also be spotted in Fig 4 (3^rd^ column, 2^nd^ row). Furthermore in line with VOI analyses, neither activation nor deactivation could be in SPM analyses for non-auditory regions (SC, CB and ON) of normal hearing animals.

Employing ABL as reference condition in SPM analyses nearly identically to VOI analyses significant activations could be observed with all other conditions (RN(95dB), WN(65dB) and BG(55dB)) in IC, OC and AC. The only exception was a lack of activation in OC due to BG(55) condition. This can also be extracted from Fig 4, showing activations with all conditions (RN(95dB), WN(65dB) and BG(55dB)) in IC and AC (column 4–6, row 1–2), and in OC only with RN(95dB) and WN(65dB) but not with BG(55dB) according to the VOI atlas (column 4–6, row 4). Significant activations in the above three regions were at a considerably higher level (according to T_max_-fractions) with ABL compared to BG(55dB) reference–e.g. for the IC and RN(95dB) stimulation 4.70 vs. 2.66.

Moreover, SPM analyses with ABL reference did not reveal any activation in MGB or NC. With respect to non-auditory regions—again in line with VOI analyses—SPM detected significantly higher activity in ON in ABL condition compared to all acoustic conditions in healthy rats (T_max_-fractions -1.86 to -2.73). SPMs displayed in Fig 4 show, that these “activations” due to ABL condition (recognizable in blue as “deactivations” during stimulation) considerably exceed the ON into the frontal cortex (column 4–6, rows 1–3).

## Discussion

### Activation of the auditory system in normal hearing rats and differences to humans

Using ^18^F-FDG PET, we were able to show activations in several anatomical structures along the central auditory pathway of normal hearing rats. Likewise Jang et al. [13] employed ^18^F-FDG PET to study stimulus-related changes of activity in the same regions of the central auditory pathway as we did. However in their study white noise stimuli at different sound pressure levels between 40 dB (for reference) and 100 dB (strongest stimulus) were applied. This type of stimulus is different compared to the pulsed rippled noise used as strongest stimulus in our study. We chose a lesser sound pressure level (95 dB) as prolonged 100 dB stimulation can induce noise trauma in rats [20, 21] and shows negative effects even with short exposure [22, 23]. Nevertheless, considering results obtained with the strongest stimuli, both studies demonstrate significant activations in the NC, the OC and the IC–reaching up to 15% in the mean. Moreover, we detected a significant increase in MGB, which was reported only not-significant by Jang et al. [13].

Different results with strongest stimuli were observed for AC with no change in our study (±0%) and a significant decrease of more than -5% in the mean in Jang’s study [13]. Nonetheless, we observed a less pronounced but significant reduction of activity in the AC (-3%) with the less strong 65dB white noise stimulus similar to Jang’s observation at 80dB [13]. These findings of reduced activity in the AC due to white noise stimulation might be explained by adaptation to this uniform stimulus presented in both studies for 30 min [24]. The lesser reduction with our lower stimulus intensity fits the intensity related reduction observed by Jang et al. [13]. The lack of reduced activity in AC due to stimulation with the more complex pulsed rippled noise used by us is most likely due to the nature of this stimulus. It is characterized by temporal and spectral modulations varying randomly through time (representative for a multi-component signal) in a way that matches aspects of animal vocalization. Therefore adaptation effects are to be expected in a lesser amount [16]. Besides that, the observation of activations in subcortical structures (NC, OC, IC) in contrast to AC (made in our and Jang’s study) despite using white noise stimuli might be explained by the fact that synaptic depression by habituation mostly occurs in cortical areas [25].

On the other side, the lack of AC activation in rats might, in addition, have species-specific reasons. In humans, activation of the AC due to different and not specifically adaptation-avoiding stimuli presented during ^18^F-FDG uptake phase has been demonstrated [26–28]. Furthermore one might speculate that limitations in demonstrating AC activation in rats (and subcortical structures in humans) can be related to the respective sizes of these structures in relation to the spatial resolution of the respective PET equipment. The diameter of AC in humans (≈ 50 mm) equals approximately 7-times the spatial resolution (FWHM ≈ 7 mm) while in rats (AC ≈ 3.2 mm) it is only 2-times (FWHM ≈ 1.5 mm) [10, 12, 29, 30]. Therefore, the expected loss of signal from AC due to partial volume effects is much higher in rats compared to humans [31]. Vice versa, the diameter of subcortical structures like the MGB in rats (≈ 1.5 mm) equals approximately at least the spatial resolution, while in humans (≈ 4.5 mm) it corresponds to only 0.6-times the resolution [10, 12, 29, 30]. Consequently, the expected loss of signal from MGB and/or other subcortical structures due to partial volume effects in humans exceeds that in rats[31]. In fact, the recovery of signal from activation of subcortical auditory structures in humans seems to be so low that it has only rarely been reported by now [6], which is the strength of using animal models. Nevertheless, it should in general be possible to detect activation in subcortical structures in humans much better using the most recent PET technology with a spatial resolution approaching the diameter of these structures [32].

### Findings after cochlea ablation in rats

Effects of cochlea ablation on activity in the central auditory pathway and cortex have been previously studied using ^14^C-2-Deoxyglucose (2-DG) ex-vivo autoradiography [33] and ^18^F-FDG PET [8]. Ahn et al. [33] performed bilateral cochlea ablation in rats and injected 2-DG under ambient noise. They observed a significant reduction of 2-DG uptake (compared to normal rats) in AC at 2 and 8 weeks post-ablation and IC at 2 and 4 weeks post-ablation–more pronounced in the latter region–and no significant change at 1 and 28 weeks post-ablation. Furthermore, in the MGB significant reduction was only found 2 weeks postoperatively. Our finding of significantly lower ^18^F-FDG uptake in AC and IC together with no significant change in MGB four weeks after ablation compared to measurements during background noise condition preoperatively correspond to the changes observed in these regions by Ahn et al. [33]. In our study uptake reductions were most pronounced in the IC as well. In addition, we could demonstrate reduced activity in the OC after cochlea ablation. Hsu et al. [8] compared after unilateral cochlea ablation ipsilateral and contralateral activity in AC and IC–during ambient noise condition and stimulation 50–60 dB SPL above the ambient noise (i.e. with 115 dB). Ratios expressing activity during ambient noise on the side mainly associated with the preserved cochlea against activity on the side mainly associated with the ablated cochlea equaled 1.24±0.08 for the IC and 1.18±0.07 for the AC. Acoustic stimulation increase activity in the IC to 1.36±0.14 but not in the AC. These results are basically well in line with our present results. Likewise, we observed (compared to cochlea ablated status) higher activity due to ambient noise (BG(55dB)) preoperatively in the IC as compared to the AC (18±4% vs. 10±2%) and an increase due to auditory stimulation (RN(95dB)) for the IC (35±4%) but not for the AC (10±3%). SC and CB as reference regions showed (as expected) no significant change due to auditory stimulation and after cochlea ablation. On the contrary, an increase in activity of more than 20% after ablation in comparison to each of the three auditory conditions preoperatively was seen in the ON. Such extra-auditory effects were not described in the studies of Ahn et al. [33] and Hsu et al. [8]. It is known particularly for the visual and auditory system that deprivation of sensory input to one sense can lead to cross-modal neuronal plastic changes with enhanced activity in cortex region primarily belonging to the other sense [34, 35]. With respect to the olfactory system, a regulatory impact of the auditory system via a noradrenergic cortical modulation on the olfactory system has been demonstrated in a learning paradigm [36]. Furthermore, based on behavior tasks and local field potential recordings, Zhou et al. [37] showed that cross-modal enhancement of olfactory perception was present 7 days after visual deprivation. We observed this analogously in our study while testing in a dark environment. A corresponding effect of higher olfactory activity about 22% after cochlea ablation was found.

### Limitations

As ^18^F-FDG PET is performed as an integrative measure over 30min, detection probability of cortical activation of small regions, such as single cells, or with low frequency of activation is only limited. Visualization of information transmission by sparse coding as discussed for the AC [38] might not be possible with ^18^F-FDG-PET.

Furthermore, use of standard VOI atlases in analyses might result in misalignment and low coverage due to the variability among animals even of the same breed. For small regions, this could cause loss of detected activations which are identifiable in SPM by visual assessment. In our study, the SPM results displayed in Fig 4 (column 6, row 4) shows an activation which could be located in the OC. Nevertheless, we limited our analyses to the standard VOI atlas in order to be comparable to established methods in humans.

We decided to selected hypothesis-driven representative control regions–ON and CB as non-cortical control and SC as cortical control–instead of data-driven VOI regions.

Even though constant acoustic, climatic and olfactory conditions have been maintained, a small possibility remains that changes in the olfactory system have been influenced by hidden changes in the environment over the duration from January to May for example due to seasonal clothing. We expect any hidden impact to be negligible compared to the effect caused by ablation.

## Conclusions

This study demonstrates the usefulness of small animal PET with ^18^F-FDG to image auditory system activations along nuclei of the central auditory pathway in normal hearing rats. For the strongest, non-adaptive stimulus (95dB, rippled noise) against background noise (55 dB) activations were consistently seen in NC, OC, MGB and IC with VOI and voxel-based analyses. A functional deactivation of the AC was measurable comparing a continuous white noise to both previous mentioned auditory conditions. Nevertheless, our data indicate, that sound deprivation to the greatest possible extent is necessary for the reference condition to allow for detection of increased activation in the AC even when a strong non-adaptive stimulus is used for stimulation. Furthermore, we found evidence for neuronal plasticity following continuous complete sound deprivation (cochlea ablation) in the form of increase (compensatory) activity in the olfactory system. The specificity of auditory and cross-modal compensatory activations is supported by the fact that no activations were detected in any reference non-auditory region (SC, CB). In summary, small animal ^18^F-FDG PET appears to be very promising to evaluate the pathophysiology of hearing loss in rats and the process of hearing restoration following auditory / cochlear implantation. Due to the minimal invasive procedures of PET imaging, the combinability of ^18^F-FDG imaging with other additional PET tracers, e.g. for different neurotransmitters [39], further enables a more-extensive observation and characterization of hearing, hearing disorders and corresponding healing processes. Moreover, the applicability of PET in medium size animals and humans makes it a valuable tool for translational hearing research.

## Supporting information

S1 Table ANCOVA with FDG uptake as the dependent variable. Results of ANCOVA with the condition, the order of condition and animals as well as anatomical area as fixed effects–uptake in the PET scan as dependent variable and animal as a random effect (performed using JMP10 software). ANCOVA was performed without the ablation data as stimulation conditions are only possible before ablation.
(DOCX)
