# Supplementary material for: Visualization of the auditory pathway in rats with 18F-FDG PET activation studies based on different auditory stimuli and reference conditions including cochlea ablation
Source: PLoS One. 2018 Oct 2;13(10):e0205044. doi: 10.1371/journal.pone.0205044 (PMC6168174; doi:10.1371/journal.pone.0205044)
Supplement: S1 Table — Results of ANCOVA with the condition, the order of condition and animals as well as anatomical area as fixed effects–uptake in the PET scan as dependent variable and animal as a random effect (performed using JMP10 software). ANCOVA was performed without the ablation data as stimulation conditions are only possible before ablation. (DOCX) [file pone.0205044.s001.docx]

S1 Table: ANCOVA of study with FDG uptake as the dependent variable

| **Source** | **Degrees of Freedom** | **F Ratio** | **Prob. > F** |
| --- | --- | --- | --- |
| Condition | 2 | 15.8442 | < 0.0001 |
| Order of Condition | 2 | 0.5685 | 0.5670 |
| Animal position | 2 | 0.5604 | 0.5716 |
| Anatomical area | 8 | 387.2309 | < 0.0001 |

Results of ANCOVA with the condition, the order of condition and animals as well as anatomical area as fixed effects – uptake in the PET scan as dependent variable and animal as a random effect (performed using JMP10 software). ANCOVA was performed without the ablation data as stimulation conditions are only possible before ablation.
